# Supplementary material for: Dementia and the risk of short-term readmission and mortality after a pneumonia admission
Source: PLoS One. 2021 Jan 28;16(1):e0246153. doi: 10.1371/journal.pone.0246153 (PMC7842970; doi:10.1371/journal.pone.0246153)
Supplement: S1 Appendix — aHighest level of education was classified according to the UNESCO classification as low (< 10 years), middle (10–15 years), and higher education (> 15 years). Due to unknown level of education in the earlier calendar period, this variable was limited to the last calendar period (i.e. 2012–2016). (DOCX) [file pone.0246153.s001.docx]

**Socioeconomic factors**

| **S1 Appendix: Information on socioeconomic factors obtained from the Civil Registration System and Statistics Denmark** | |
| --- | --- |
| **Cohabitation status** |  |
| Living with a partner |  |
| Married |  |
| Registered partnership |  |
| Cohabitation |  |
| Living alone |  |
| **Highest level of education, years^a^** |  |
| > 15 |  |
| 10-15 |  |
| < 10 |  |
| ^a^Highest level of education was classified according to the UNESCO classification as low (< 10 years), middle (10–15 years), and higher education (> 15 years). Due to missing information on highest level of education in the earlier calendar period, this variable was limited to the last calendar period (ie 2012-2016). | |
