# Supplementary material for: Dementia and the risk of short-term readmission and mortality after a pneumonia admission
Source: PLoS One. 2021 Jan 28;16(1):e0246153. doi: 10.1371/journal.pone.0246153 (PMC7842970; doi:10.1371/journal.pone.0246153)
Supplement: S2 Appendix — aAssessment of residency in nursing home in the month preceding index admission. Nursing homes are for permanent residents, and the nursing homes are staffed throughout the day by health care professionals. bAssessment of home care in the month preceding index admission. Home care is delivered to individuals living at home who are unable to manage everyday life on their own. (DOCX) [file pone.0246153.s002.docx]

**Type of residency and home care**

| **S2 Appendix: Information on type of residency and home care services obtained from Statistics Denmark** | |
| --- | --- |
| **Type of residency^a^** | |
| Nursing home |  |
| Not at nursing home |  |
| **Home care^b^** | |
| Personal care |  |
| Independent (no home care) | |
| Dependent (any extent of personal home care) | |
| Practical help  Independent (no home care)  Dependent (any extent of practical home care) | |
| ^a^Assessment of residency in nursing home in the month preceding index admission. Nursing homes are for permanent residents, and the nursing homes are staffed throughout the day by health care professionals.  ^b^Assessment of home care in the month preceding index admission. Home care is delivered to individuals living at home who are unable to manage everyday life on their own. | |
