# Supplementary material for: Dementia and the risk of short-term readmission and mortality after a pneumonia admission
Source: PLoS One. 2021 Jan 28;16(1):e0246153. doi: 10.1371/journal.pone.0246153 (PMC7842970; doi:10.1371/journal.pone.0246153)
Supplement: S3 Appendix — aModified from original index by removing the categories “Thyroid disorder” and “Gout”. bModified from original index by removing the category”Allergy”. cModified from original index by adding the category “Dysphagia” (ICD-10: R13). dModified from original index by removing the ATC code N02A. eModified from original index by removing the category “HIV/AIDS” and renamed “Anemias”. fModified from original index by removing the categories “Vision problem”, “Hearing problem”, and “Migraine”. gModified from the original index by including epilepsy diagnoses only in the definition of the category “Epilepsy”. hModified from original index by removing the category “Anorexia/bulimia” and “Dementia” (which is the exposure in this study). iModified from original index by removing N06AX12, which is used for smoking cessation. (DOCX) [file pone.0246153.s003.docx]

**Comorbidity**

| **S3 Appendix: Information on comorbidity by the Multimorbidity Index obtained from the Danish National Patient Register, the Danish Psychiatric Central Register, and the Danish National Prescription Registry** | | | | | |
| --- | --- | --- | --- | --- | --- |
| **Category** | **Coding definition** | **Diagnosis codes (ICD-10)** | **Diagnosis time frame** | **Drug codes (ATC)** | **Prescription time frame** |
| **Circulatory system** |  |  |  |  |  |
| Hypertension | Diagnosis AND/OR prescriptions of antihypertensives if not ischemic heart disease or heart failure (or kidney disease: only diuretics) | I10-I13, I15 | Since 1995 | C02, C04, C07, C08, C09, C03 | Twice last year |
| Dyslipidemia  Ischemic heart disease | Diagnosis AND/OR drug prescription for lipid-lowering drugs if not ischemic heart disease  Diagnosis AND/OR prescriptions for antianginal drug | E78  I20-I25 | Last two years  Since 1995 | C10  C01DA | Twice last year  Twice last year |
| Atrial fibrillation | Diagnosis | I48 | Since 1995 |  |  |
| Heart failure | Diagnosis | I50 | Since 1995 |  |  |
| Peripheral artery occlusive diseases | Diagnosis | I70-I74 | Since 1995 |  |  |
| Stroke | Diagnosis | I60-I64, I69 | Since 1995 |  |  |
| **Endocrine system^a^** |  |  |  |  |  |
| Diabetes mellitus | Diagnosis AND/OR prescription of antidiabetics | E10-E14 | Since 1995 | A10A, A10B | Twice last year |
| **Pulmonary system^b^** |  |  |  |  |  |
| Chronic pulmonary disease | Prescriptions for obstructive airway disease drugs |  | Since 1995 | R03 | Twice last year |
| **Gastrointestinal system** |  |  |  |  |  |
| Ulcer/chronic gastritis  Dysphagia**^c^**  Chronic liver disease  Inflammatory bowel disease  Diverticular disease of intestine | Diagnosis  Diagnosis  Diagnosis  Diagnosis  Diagnosis | K221, K25-K28, K293-K295  R13  B16-B19, K70-K74, K766, I85  K50-K51  K57 | Since 1995  Since 1995  Since 1995  Since 1995  Since 1995 |  |  |
| **Urogenital system** |  |  |  |  |  |
| Chronic kidney disease  Prostate disorders | Diagnosis  Diagnosis AND/OR prescription of prostate hyperplasia therapy drug | N03, N11, N18-N19  N40 | Since 1995  Since 1995 | C02CA, G04C | Twice last year |
| **Musculoskeletal system** |  |  |  |  |  |
| Connective tissue disorders | Diagnosis | M05-M06, M08-M09, M30-M36, D86 | Since 1995 |  |  |
| Osteoporosis | Diagnosis AND/OR prescription for osteoporosis drugs | M80-M82 | Since 1995 | M05B, G03XC01, H05AA | Twice last year |
| Painful condition**^d^** | Repeated prescriptions of analgesics |  | Since 1995 | N02BA51, N02BE, M01A, M02A | Four times last year |
| **Anemias^e^** |  |  |  |  |  |
| Anemias | Diagnosis | D50-D53, D55-D61, D63-D64 | Last two years |  |  |
| **Cancers** |  |  |  |  |  |
| Cancer | Diagnosis | C00-C43, C45-C97 | Last five years |  |  |
| **Neurological system**^f^ |  |  |  |  |  |
| Multiple sclerosis  Parkinson’s disease | Diagnosis  Diagnosis | G35  G20-G22 | Since 1995  Since 1995 |  |  |
| Epilepsy**^g^**  Neuropathies | Diagnosis AND prescriptions of anti-epileptics  Diagnosis | G40-G41  G50-G64 | Since 1995  Last two years | N03 | Twice last year |
| **Mental health**  **Conditions^h^** |  |  |  |  |  |
| Mood, stress or anxiety-related disorder | Diagnosis | F32-F34, F40-F48 | Last two years |  |  |
| Psychological distress  Alcohol problems | Prescription of antidepressants if not other mental disorder  Diagnosis | F101-F109 | Last two years | N06A^i^ | Twice last year |
| Substance abuse | Diagnosis | F11-F16, F18-F19 | Last two years |  |  |
| Bipolar affective disorder | Diagnosis AND/OR prescriptions of lithium salts | F30-F31 | Since 1995 | N05AN | Twice last year |
| Schizophrenia/skizoaffec-tive disorder | Diagnosis | F20, F25 | Since 1995 |  |  |
| ^a^Modified from original index by removing the categories “Thyroid disorder” and “Gout”.  ^b^Modified from original index by removing the category ”Allergy”.  ^c^Modified from original index by adding the category “Dysphagia” (ICD-10: R13).  ^d^Modified from original index by removing the ATC code N02A.  ^e^Modified from original index by removing the category “HIV/AIDS” and renamed “Anemias”.  ^f^Modified from original index by removing the categories “Vision problem”, “Hearing problem”, and “Migraine”.  ^g^Modified from the original index by including epilepsy diagnoses only in the definition of the category “Epilepsy”.  ^h^Modified from original index by removing the category “Anorexia/bulimia” and “Dementia” (which is the exposure in this study).  ^i^Modified from original index by removing N06AX12, which is used for smoking cessation. | | | | | |
