# Supplementary material for: Dementia and the risk of short-term readmission and mortality after a pneumonia admission
Source: PLoS One. 2021 Jan 28;16(1):e0246153. doi: 10.1371/journal.pone.0246153 (PMC7842970; doi:10.1371/journal.pone.0246153)
Supplement: S4 Appendix — aRemaining medication available in the data set, not already adjusted for in the Comorbidity Index (S3 Appendix). (DOCX) [file pone.0246153.s004.docx]

**Medication use**

| **S4 Appendix: Information on** **medication use obtained from the Danish National Prescription Registry^a^** |
| --- |

| Name | ATC code | Prescription time frame |
| --- | --- | --- |
| **Antibacterials for systemic use** | J01 | Once last six months |
| **Drugs for cardiovascular diseases** |  |  |
| Antithrombotic drugs | B01 | Once last six months |
| **Immunosuppressants** |  |  |
| Glucocorticoids | H02AB | Once last six months |
| Antineoplastic agents | L01 | Once last six months |
| Immunosuppressants | L04 | Once last six months |
| **Psycholeptics** |  | |
| Antipsychotics  Benzodiazepines  Benzodiazepine derivates  Benzodiazapine related drugs | N05A  N05BA, N05CD  N05CF | Once last six months  Once last six months  Once last six months |
| **Drugs for pain** |  |  |
| Opioids | N02A | Once last six months |

^a^Remaining medication available in the data set, not already adjusted for in the Comorbidity Index (S3 Appendix).
