# Supplementary material for: Dementia and the risk of short-term readmission and mortality after a pneumonia admission
Source: PLoS One. 2021 Jan 28;16(1):e0246153. doi: 10.1371/journal.pone.0246153 (PMC7842970; doi:10.1371/journal.pone.0246153)
Supplement: S5 Appendix — aIf a pneumonia diagnosis was registered as both a primary and a secondary diagnosis during the same admission, it was categorized as a primary diagnosis. (DOCX) [file pone.0246153.s005.docx]

**Index admission**

| **S5 Appendix: Information on index admission obtained from the Danish National Patient Register** | |
| --- | --- |
| **Pneumonia diagnosis** | |
| Primary diagnosis^a^ |  |
| Secondary diagnosis | |

^a^If a pneumonia diagnosis was registered as both a primary and a secondary diagnosis during the same admission, it was categorized as a primary diagnosis.
