# Supplementary material for: Dementia and the risk of short-term readmission and mortality after a pneumonia admission
Source: PLoS One. 2021 Jan 28;16(1):e0246153. doi: 10.1371/journal.pone.0246153 (PMC7842970; doi:10.1371/journal.pone.0246153)
Supplement: S1 Table — Abbreviations: aMRR: adjusted mortality rate ratio; CI: confidence interval. aAll analyses were adjusted for sex, age, calendar period, cohabitation status, length of stay, type of pneumonia diagnosis, time since discharge, non-psychiatric comorbidities, psychiatric comorbidities, and alcohol/substance abuse. (DOCX) [file pone.0246153.s006.docx]

**S1 Table. Adjusted mortality rate ratios (aMRRs) for the risk of 30-day mortality in pneumonia patients with dementia (defined in five different ways) versus those without dementia in 298,872 admissions**

|  | Model 3^a^  aMRR (95% CI) |
| --- | --- |
| **Without dementia** | 1 |
| **With dementia** |  |
| Main analysis | 2.29 (2.21-2.37) |
| Dementia identified only by diagnosis (ie a secondary healthcare contact) (N=15,707) | 2.22 (2.12-2.32) |
| Dementia identified only by an anti-dementia prescription (ie no diagnosis of dementia in secondary health care) (N=1,302) | 1.78 (1.53-2.07) |
| Dementia identified by both a dementia diagnosis and an anti-dementia prescription (N=8,939) | 2.50 (2.37-2.64) |
| Dementia diagnosed above the age of 60 years (N=25,374) | 2.31 (2.23-2.40) |
| Dementia diagnosed within five years prior to the index admission (N=23,186) | 2.27 (2.18-2.35) |

Abbreviations: aMRR: adjusted mortality rate ratio; CI: confidence interval

^a^All analyses were adjusted for sex, age, calendar period, cohabitation status, length of stay, type of pneumonia diagnosis, time since discharge, non-psychiatric comorbidities, psychiatric comorbidities, and alcohol/substance abuse.
