# Supplementary material for: Dementia and the risk of short-term readmission and mortality after a pneumonia admission
Source: PLoS One. 2021 Jan 28;16(1):e0246153. doi: 10.1371/journal.pone.0246153 (PMC7842970; doi:10.1371/journal.pone.0246153)
Supplement: S2 Table — Abbreviations: aMRR: adjusted mortality rate ratio; CI: confidence interval, AP: attributable proportion. aAdjusted for sex, age, calendar period, cohabitation status, length of stay, type of pneumonia diagnosis, time since discharge, somatic comorbidities, psychiatric comorbidities, and alcohol/substance abuse. bAttributable proportion was calculated as: AP = (aMRR(dementia+medication+)−aMRR(dementia+ medication-)−aMRR(dementia- medication+) + 1) / aMRR(dementia+ medication+). cExcess number of events due to interaction was calculated as Nexcess = Number of events(dementia+ medication+) * AP. (DOCX) [file pone.0246153.s007.docx]

**S2 Table. Adjusted mortality rate ratios (aMRRs) for the risk of 30-day mortality in pneumonia patients with dementia (without medication use), with use of benzodiazepines, opioids or anti-psychotics (at least one prescription within the preceding four months) (without dementia), or with both dementia and medication use, versus those with neither exposure in 298,872 admissions**

|  | **Dementia** | | | | Attributable proportion due to interaction^b^ (95% CI) | Excess number of events due to interaction^c^  (95% CI) |
| --- | --- | --- | --- | --- | --- | --- |
|  | **No** | | **Yes** | |  |  |
|  | **Medication** | | **Medication** | |  |  |
|  | **No**  aMRR^a^  (95% CI) | **Yes**  aMRR^a^  (95% CI) | **No**  aMRR^a^  (95% CI) | **Yes**  aMRR^a^  (95% CI) |  |  |
| Benzodia-zepines | 1 | 1.21  (1.17; 1.25) | 2.38  (2.28; 2.47) | 2.53  (2.36; 2.69) | -0.02  (-0.10; 0.05) | -25  (-104; 54) |
| Opioids | 1 | 1.57  (1.52; 1.62) | 2.53  (2.43; 2.64) | 2.99  (2.81; 3.17) | -0.04  (-0.10; 0.03) | -53  (-145; 39) |
| Antipsy-chotics | 1 | 1.76  (1.66-1.85) | 2.14  (2.05; 2.22) | 3.40  (3.19; 3.61) | 0.15  (0.09; 0.21) | 194  (117; 271) |

Abbreviations: aMRR: adjusted mortality rate ratio; CI: confidence interval, AP: attributable proportion.

^a^Adjusted for sex, age, calendar period, cohabitation status, length of stay, type of pneumonia diagnosis, time since discharge, somatic comorbidities, psychiatric comorbidities, and alcohol/substance abuse.

^b^Attributable proportion was calculated as: AP = (aMRR_(dementia+medication+)_ – aMRR_(dementia+ medication-)_ – aMRR_(dementia- medication+)_ + 1) / aMRR_(dementia+ medication+)_

^c^Excess number of events due to interaction was calculated as N_exccess_ = Number of events_(dementia+ medication+)_ * AP
