# Supplementary material for: Dementia and the risk of short-term readmission and mortality after a pneumonia admission
Source: PLoS One. 2021 Jan 28;16(1):e0246153. doi: 10.1371/journal.pone.0246153 (PMC7842970; doi:10.1371/journal.pone.0246153)
Supplement: S4 Table — Abbreviations: aIRR: adjusted incidence rate ratio; CI: confidence interval, AP: attributable proportion. aAdjusted for sex, age, calendar period, cohabitation status, length of stay, type of pneumonia diagnosis, time since discharge, somatic comorbidities, psychiatric comorbidities, and alcohol/substance abuse. bAttributable proportion was calculated as: AP = (aIRR(dementia+ medication+)−aIRR(dementia+ medication-)−aIRR(dementia- medication+) + 1) / aIRR(dementia+ medication+). cExcess number of events due to interaction was calculated as Nexcess = Number of events(dementia+ medication+) * AP. (DOCX) [file pone.0246153.s009.docx]

**S4 Table. Adjusted incidence rate ratios (aIRRs) for the risk of 30-day readmission in pneumonia patients with dementia (without medication use), with use of benzodiazepines, opioids or anti-psychotics (at least one prescription within the preceding four months) (without dementia), or with both, versus those with neither exposure in 298,872 admissions**

|  | **Dementia** | | | | Attributable proportion due to interaction^b^ (95% CI) | Excess number of events due to interaction^c^  (95% CI) |
| --- | --- | --- | --- | --- | --- | --- |
|  | **No** | | **Yes** | |  |  |
|  | **Medication** | | **Medication** | |  |  |
|  | **No**  aIRR^a^  (95% CI) | **Yes**  aIRR^a^  (95% CI)^a^ | **No**  aIRR^a^  (95% CI)^a^ | **Yes**  aIRR^a^  (95% CI)^a^ |  |  |
| Benzodia-zepines | 1 | 1.12  (1.10; 1.14) | 1.09  (1.05; 1.12) | 1.16  (1.09; 1.22) | -0.04  (-0.11; 0.02) | -60  (-151; 31) |
| Opioids | 1 | 1.22  (1.20; 1.25) | 1.12  (1.08; 1.16) | 1.19  (1.13; 1.25) | -0.13  (-0.19; -0.06) | -210  (-316; -103) |
| Antipsy-chotics | 1 | 1.09  (1.05; 1.13) | 1.07  (1.04 1.10) | 1.11  (1.04; 1.17) | -0.05  (-0.12; 0.02) | -62  (-155; 30) |

Abbreviations: aIRR: adjusted incidence rate ratio; CI: confidence interval, AP: attributable proportion

^a^Adjusted for sex, age, calendar period, cohabitation status, length of stay, type of pneumonia diagnosis, time since discharge, somatic comorbidities, psychiatric comorbidities, and alcohol/substance abuse.

^b^Attributable proportion was calculated as: AP = (aIRR_(dementia+ medication+)_ – aIRR_(dementia+ medication-)_ – aIRR_(dementia- medication+)_ + 1) / aIRR_(dementia+ medication+)._

^c^Excess number of events due to interaction was calculated as N_exccess_ = Number of events_(dementia+ medication+)_ * AP
