# Supplementary material for: Dementia and the risk of short-term readmission and mortality after a pneumonia admission
Source: PLoS One. 2021 Jan 28;16(1):e0246153. doi: 10.1371/journal.pone.0246153 (PMC7842970; doi:10.1371/journal.pone.0246153)
Supplement: S6 Table — Abbreviations: aIRR: adjusted incidence rate ratio; CI: confidence interval. aMain analysis was restricted to the years 2012–2016 and adjusted for sex, age, calendar period, cohabitation status, length of stay, type of pneumonia diagnosis, time since discharge, somatic comorbidities, psychiatric comorbidities, and alcohol/substance abuse. bFurther adjusted for educational level, type of residency, and extent of home care. (DOCX) [file pone.0246153.s011.docx]

**S6 Table. Adjusted incidence rate ratios (aIRRs) for the risk of 30-day readmission in pneumonia patients with dementia versus those without dementia during 2012-2016 in 106,949 admissions**

|  | Model 3^a^  aIRR (95% CI) | Model 3a^b^  aIRR (95% CI) |
| --- | --- | --- |
| Without dementia | 1 | 1 |
| With dementia | 1.07 (1.03; 1.12) | 1.08 (1.03; 1.13) |

Abbreviations: aIRR: adjusted incidence rate ratio; CI: confidence interval

^a^Main analysis was restricted to the years 2012-2016 and adjusted for sex, age, calendar period, cohabitation status, length of stay, type of pneumonia diagnosis, time since discharge, somatic comorbidities, psychiatric comorbidities, and alcohol/substance abuse.

^b^Further adjusted for educational level, type of residency, and extent of home care.
